# Ceramides and the Defective Barrier in Atopic Dermatitis

**DOI:** 10.1159/spp/aetag001

**Published:** 2026-07-29

**Authors:** Philip W. Wertz, Joachim W. Fluhr

**Affiliations:** a Oral Pathology, Radiology and Medicine, University of Iowa, Iowa City, IA, USA; b Charite–Universitatsmedizin Berlin, Institute of Allergology IFA, Berlin, Germany

**Keywords:** Atopic dermatitis, Barrier function, Ceramides, Stratum corneum, *Staphylococcus aureus*

## Abstract

**Background:**

Atopic dermatitis (AD) is a common remitting-relapsing inflammatory skin disease characterized by eczematous lesions, xerosis, and pruritus. It is associated with a defective permeability barrier and increased susceptibility to *Staphylococcus aureus* colonization. It has been demonstrated that the abrogated epidermal barrier is related to a decreased mass of ceramides and long-chain fatty acids in the stratum corneum (SC). The inherent pH of healthy SC is below 5, and a low pH is required for proper assembly of the barrier lipids. In AD, the pH is elevated.

**Summary:**

A defective permeability barrier of the SC is the primary defect in AD. This defect reflects a reduction of ceramide and fatty acid mass in the SC due to reduced glucocerebrosidase and acid sphingomyelinase activity and a shift in fatty acid synthesis from long-chain to short-chain fatty acids and the incorporation of these shorter fatty acids into ceramides. Topically applied ceramides can restore epidermal function to a damaged barrier. Ceramide-containing moisturizers have produced partial restoration of the barrier function in AD by influencing lipid organization. Other topical and systemic therapies are also discussed.

**Key Message:**

Correcting the barrier defect by topically supplied ceramides and long-chain fatty acids in sufficient amounts could contribute to restoration of the microbiome and decreasing irritation and pruritus.

## Introduction

The composition and structure of the permeability barrier provided by the stratum corneum (SC) of the skin has been reviewed [1–4]. The number of cell layers in human SC of healthy skin varies regionally; however, most regions of the body, including the volar forearm, have between 15 and 25 layers of corneocytes [5, 6]. The volar forearm is the site most frequently used for transepidermal water loss (TEWL) measurements in studies of atopic dermatitis (AD). The dominant cells of the SC, corneocytes, are flattened cells containing keratin filaments and a high concentration of amino acids, urocanic acid, pyrrolidone carboxylic acid, and lactic acid [7, 8]. These small molecules come from filaggrin degradation and anaerobic glycolysis and are collectively called natural moisturizing factor (NMF) [8]. The high concentration of NMF causes the internal compartment of the corneocyte to have a high osmotic strength, which helps retain water. The corneocytes are bounded by a layer of cross-linked protein known as the cornified envelope. The outer surface of the cornified envelope is coated by a covalently bound layer of ω-hydroxy ceramides [9–13]. This layer, the corneocyte lipid envelope, is essential for barrier function of the skin and is thought to serve as a scaffold upon which the intercellular lipid lamellae organize [13, 14]. In healthy skin, ceramides, free fatty acids and cholesterol form multilamellar structures within the intercellular spaces of the SC. These lipids form mainly trilamellar structures with a thickness of 13 nm [13–16]. This long periodicity phase (LPP) has been found in normal human SC by transmission electron microscopy and by X-ray diffraction [14, 16]. The LPP has also been found in SC from mice and pigs [17, 18]. A minor short periodicity phase of about 6 nm is also present. The LPP appears to be a trilamellar structure [19, 20]. One study of reconstituted lipids using an EOS analog suggests that the LPP should not exist in the SC [21]. This study is at odds with other studies using model SC lipids [2]. The lateral chain packing of the lipids in healthy SC is predominantly orthorhombic with a lesser amount of hexagonal chain packing [2].

In healthy SC, ceramides, cholesterol, and fatty acids represent about 50%, 27%, and 12% of the lipid mass, respectively [15]. Cholesterol sulfate and cholesterol esters make up the remainder. There are 12 major structural classes of free ceramides and 4 classes of covalently bound ceramides in SC [2–4]. In a ceramide nomenclature system, the amide-linked fatty acid is designated an N for a non-hydroxy fatty acid, A for an α-hydroxy acid, and O for an ω-hydroxy acid [22]. The long-chain base is designated by S for sphingosine, dS for dihydrosphingosine, P for phytosphingosine, and H for 6-hydroxysphingosine. The ω-hydroxy acids are 30+ carbons long, and in the ceramides that contain ω-hydroxy acids, there is a fatty acid ester-linked to the ω-hydroxyl group. This is designated by a prefix E. The major ester-linked fatty acid is linoleate. The covalently bound ceramides are derived from the CER[EOx] ceramides. The corneocyte lipid envelope is essential for barrier function.

Ceramides are synthesized in the viable epidermis and converted to glucosylceramides and sphingomyelin [23]. These polar lipids are incorporated into lamellar granules and are delivered to the bottom of the SC, where they are converted back to ceramides. Ceramide synthases in the viable epidermis combine dihydrosphingosine with fatty acyl-CoA to produce ceramide [NdS] [24]. There are six ceramide synthases that differ primarily in fatty acyl-CoA chain length specificity. All six of the ceramide synthases can use α-hydroxy fatty acyl-CoAs to ultimately lead to ceramides [AdS], [AS], [AP], and [AH] [25]. Introduction of a double bond into the initial ceramide [NdS] produces ceramide NS. There are two desaturases designated DEGS1 and DEGS2 that can introduce the double bond in dihydroceramide to produce a sphingosine-containing ceramide. However, DEGS2 is bifunctional [26]. It introduces a double bond into the shorter fatty acyl chains, but it hydroxylates carbon-4 to produce phytosphingosine-containing ceramides from dihydroceramides-containing longer fatty acyl chains. This leads to formation of ceramides [NP], [AP], and [OP]. Hydroxylation of the 6-carbon of sphingosine produces ceramides AH and NH. Although the 6-hydroxylated ceramides are well established, the hydroxylase has not been identified [27]. When the initial fatty acyl-CoA contains ω-hydroxy acids with 28 or more carbons, a fatty acid, primarily linoleic acid, is transferred to the ω-hydroxyl group by the patatin-like phospholipase domain-containing enzyme (PNPLA1) and the EOx ceramides are produced [28]. The long-chain base can be modified as described to result in ceramides [EOdS], [EOS], [EOP], and [EOH]. The metabolic pathways for ceramide synthesis are summarized in Figure 1.

**Fig. 1.**
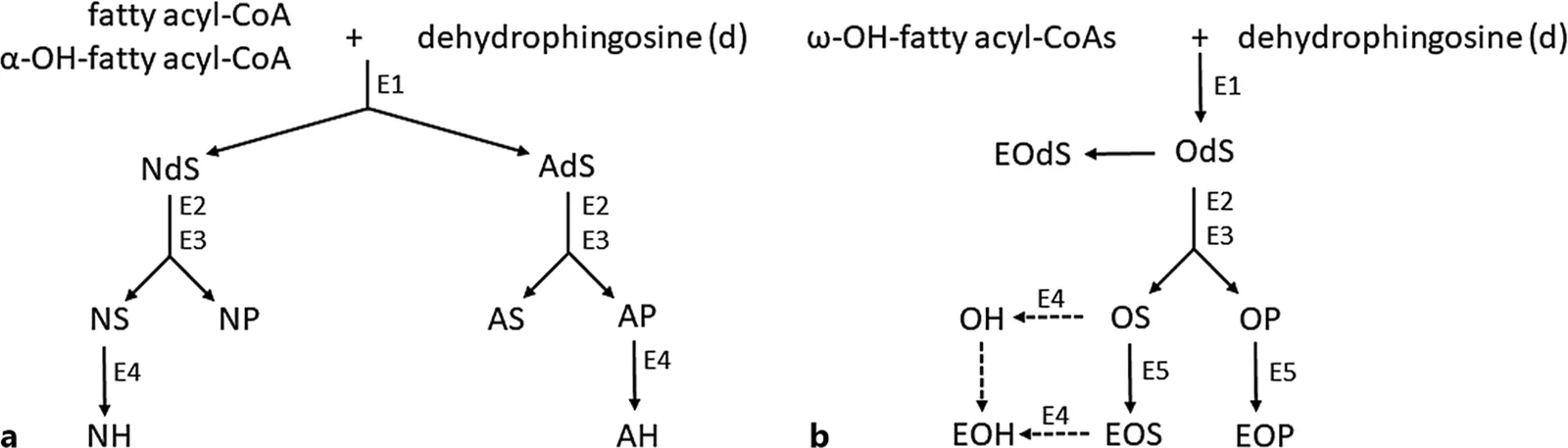
A simplified biosynthetic pathway for ceramide synthesis. Fatty acyl-CoA elongases and a 2-hydroxylase are involved in providing fatty acyl-CoA and α-hydroxy fatty acyl-CoAs of different chain lengths. The enzymes catalyzing the indicated steps are ceramide synthases 1–6 (E1), bifunctional delta-4 sphingolipid desaturases/ω-hydroxylases DEGS1 (E2) and DEGS2 (E3), a hydroxylase (E4) and patatin-like phospholipase domain-containing 1, PNPL1, (E5). **a** Synthesis of ceramides NdS, Ns, Ads, AS, NP, AP, NH, and AH. **b** Synthesis of the acylceramides EOdS, EOS, EOP, and EOH. **a** The non-acylated ceramides include non-hydroxy fatty acids and α-hydroxy fatty acids amide-linked to phytosphingosine, sphingosine, dihydrosphingosine, or 6-hydroxysphingosine: NdS, NS, AdS, NS, NP, AP, NH, and AH. **b** ω-Hydroxy ceramides and esterified ω-hydroxy ceramides (EOdS, EOS, EOP, and EOH). The enzymes are ceramide synthases 1–6 (E1), delta-4 sphingolipid desaturases DEG1 (E2) and DEGS2 (E3), hydroxylase (E4), and patatin-like phospholipase domain-containing protein 1, PNPLA1 (E5).

The EOx ceramides are also the precursors of the cornified lipid envelope [10–13]. The metabolic pathway leading from ceramide EOS to the covalently bound corneocyte lipid envelope is shown in Figure 2. The enzymes involved in this conversion were identified in studies of genetic mutations, leading to congenital recessive ichthyoses [29]. The first two steps in this pathway are catalyzed by lipoxygenases, 12R-LOX and eLOX3. The action of these two enzymes on the linoleate of acylglucosylceramide produces a hepoxilin. An epoxide hydrolase acts on the epoxide to produce a trihydro derivative that can be acted upon by an esterase to release a trihydroxy fatty acid derivative, a trioxilin. This trioxilin may have a regulatory role in keratinization. The ω-hydroxy glucosylceramide is then attached to the outer surface of the cornified envelope to form the corneocyte lipid envelope. This may be catalyzed by transglutaminase 1 [30]. Alternatively, the enzyme SDR9C7 can oxidize the hydroxyl group on carbon 13 of the linoleate to a ketone, which can form a Schiff base with free amino groups on the surface of the cornified envelope [31].

**Fig. 2.**
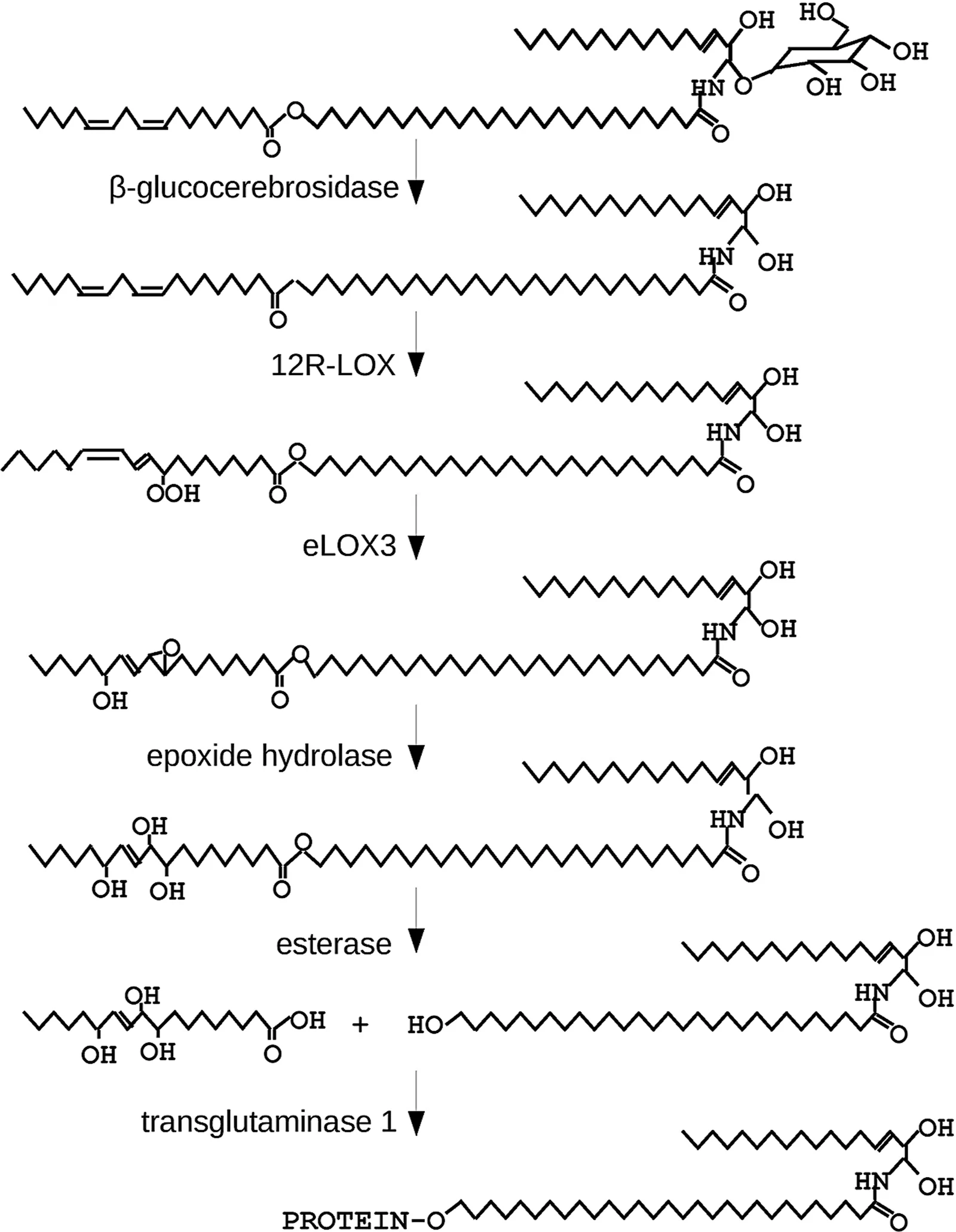
A metabolic pathway leading from GLC ceramide EOS to the cornified lipid envelope [11]. Glucosylceramide EOS is enzymatically converted into lipid species that become covalently attached to proteins of the cornified envelope. This involves the sequential actions of β-glucocerebrosidase to remove glucose, two lipoxygenases and epoxide hydrolase to modify the linoleate chain, an esterase to release the modified linoleate and finally transglutaminase 1 to attach the ω-hydroxy ceramide to the outer surface of the cornified envelope.

The intercellular lipids of the SC are organized into multiple lamellar structures between the corneocytes. In healthy skin, a major lamellar periodicity of 13 nm is called the LPP [14–16]. This reflects one trilaminar unit. The outermost lamella that can be visualized in transmission electron microscope is the corneocyte lipid envelope [9, 13–15]. An example of the intercellular lipid lamellae as revealed by transmission electron microscopy is shown in Figure 3.

**Fig. 3.**
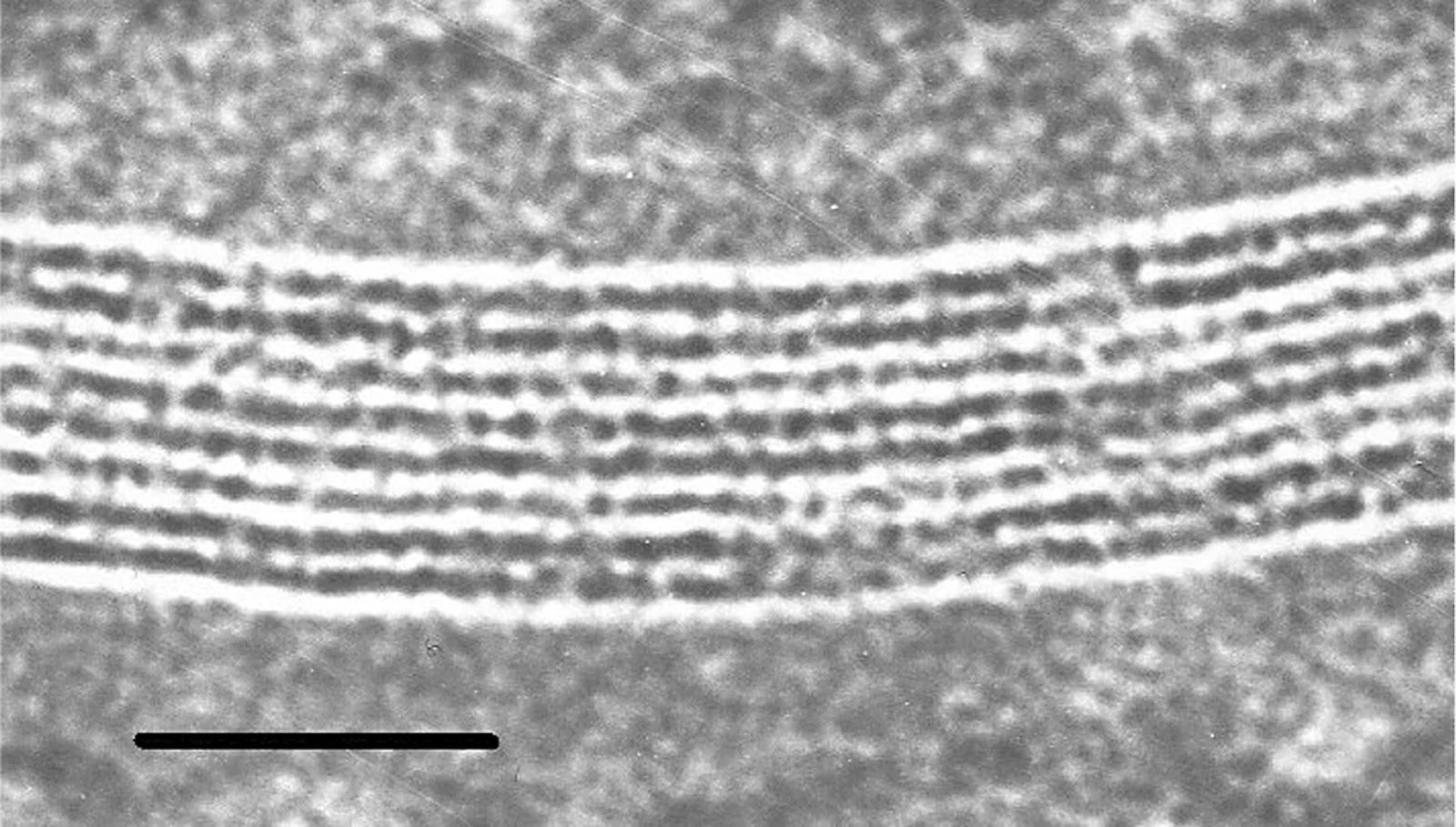
A transmission electron micrograph of human forearm skin after fixation with ruthenium tetroxide shows intercellular lipid lamellae in an intercellular space of the SC. Size bar = 50 nm. Modified from reference [15].

AD is a chronic disease characterized by dry, itchy skin with fluctuating disease activity, resulting in appearance and resolution of erythematous lesions [32]. It is one of the most common inflammatory skin diseases worldwide. It has been estimated that 15–20% of children and up to 10% of adults have AD. One estimate of the prevalence of AD in the USA puts it at 10.7% of children and 7.2% of adults. AD adversely affects the quality of life of the patient and their family members [33, 34].

The differences between the lipid compositions of healthy SC and non-lesional SC in AD are relatively small [35]; however, the absolute amounts of free fatty acids, CER[NP], CER[NH], CER[NdS], and all of the CER[EOx] are decreased in lesional SC of AD, while CER[NS], CER[AS], CER[AP], CER[AH], and CER[AdS] amounts are increased [36]. This is shown in Figure 4. The amounts of covalently bound ω-hydroxy ceramides in the corneocyte lipid envelope are reduced [10, 37]. This change in fatty acid and ceramide profile has a deleterious effect on the functionality of the skin barrier in AD.

**Fig. 4.**
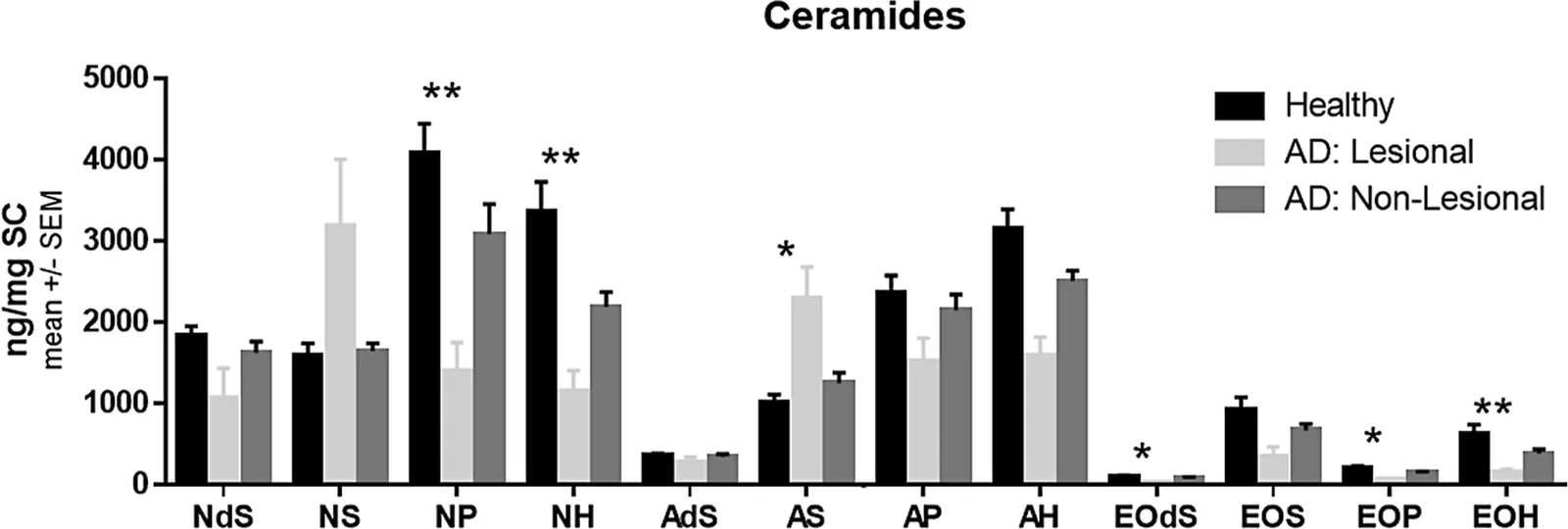
The amounts (mean +/− SEM) of ceramides in SC from healthy subjects (N = 5), from AD non-lesional SC (N = 10) and from AD lesional SC (N = 3). Based on data from [36]. An ANOVA with Kruskal-Wallis test was performed on each lipid class. In case of *p* < 0.05, a Dunn’s multiple comparisons test was performed between the healthy, lesional AD and non-lesional AD skin. Values are shown as mean +/− SEM. Only healthy vs. lesional skin showed significant differences for NP, NH, AS, EOdSS, and EOH with *p < 0.05 and **p < 0.01.

The decrease in the proportion of free fatty acids is attributed to a shift from synthesis of mostly long-chain saturated fatty acids to short-chain fatty acids. This results, at least in part, from the presence of *Staphylococcus aureus* on the skin of subjects with AD [38]. *S. aureus* induces the downregulation of the genes coding for several fatty acid elongases. This results in production of fatty acids with 14–18 carbons instead of 20–28 carbons. These shorter fatty acids are also incorporated into ceramides [39]. As a result of these structural modifications, the lamellar spacing of the SC lipid lamellae is reduced and there is a shift from predominantly orthorhombic lateral chain packing to more hexagonal chain packing. These alterations in lipid organization result in reduced permeability barrier function.

## AD Mechanisms – Inside-Out versus Outside-In

The condition of the epidermis in AD is determined by a balance between coexisting outside-in and inside-out mechanisms [40]. In the outside-in mechanism, the defective SC permeability barrier is the primary defect. This allows the penetration of airborne antigens, and these antigens trigger an adaptive immune response, leading to inflammation [41]. The inflammation leads to further impairment of the barrier. In the inside-out mechanism, the inflammation comes first and causes barrier impairment [41].

A barrier defect precedes development of overt symptoms of AD [42, 43]. The impaired barrier underlying the initiation of AD in the outside-in mechanism is largely the result of mutations in a number of genes. Most notable, in this regard, are the *SPINK5* gene coding for the serine protease inhibitor LEKTI, and the *FLG* gene coding for filaggrin [44]. Loss of function mutations in *FLG* and *SPINK5* predispose individuals to development of AD [45]. Filaggrin is important for the aggregation of keratin filaments within the corneocytes and is the major precursor of NMF. The breakdown of filaggrin to form NMF is a factor in lowering the pH of the SC. In the absence of filaggrin, the SC pH is elevated and a paracellular barrier abnormality is produced [46]. It has been demonstrated that a low pH is required for proper assembly of the SC barrier lipids in vitro [47]. Mutations in SPINK5 are associated with the early onset of AD in children up to 8 years of age, while mutations in CHI3L1, which codes for a glycosyl hydrolase, are associated with AD onset after 8 years of age [48]. More than 30 other genetic loci have been associated with AD without specific identification of the gene products [49]. Consistent with the “outside-in” hypothesis, elevated TEWL, a measure of permeability barrier function, in newborns or infants during the first week of life is predictive of subsequent development of AD [42, 43].

## Treatments Directed at the Inside-Out Mechanism

Most current treatments for AD are directed at reducing inflammation. Topical glucocorticoids are the traditional first line of treatment across all degrees of AD severity [50]. It is advisable to use the lowest potency and shortest duration to achieve clinical efficacy and to minimize side effects. Glucocorticoids act by upregulating the expression of a protein called macrocortin, which inhibits phospholipase A2 [51]. Inhibiting phospholipase A2 reduces the availability of arachidonic acid, and thereby reduces the production of inflammatory prostaglandins and leukotrienes. Glucocorticoids also cause vasoconstriction, thereby reducing edema. Glucocorticoids, depending upon the potency and the length of time used, can cause atrophy of the skin and may become less effective at treating symptoms [52, 53].

Topical calcineurin inhibitors, such as Protopic Ointment (tacrolimus) and Elidel (pimecrolimus), may be used alone or in combination with corticosteroids for treatment of mild-to-moderate AD [54]. Calcineurin is a calcium- and calmodulin-dependent serine/threonine-specific protein phosphatase [55]. Inhibition of this enzyme prevents inflammation by preventing the activation of T lymphocytes [56]. Tacrolimus and pimecrolimus both have an FDA-required boxed warning in their package inserts [57]. This was based, in part, on the post-marketing findings of malignancies in children and adults who were using these topical calcineurin inhibitors. This warning reads in part, “Long-term safety of topical calcineurin inhibitors has not been established. Although a causal relationship has not been established, rare cases of malignancy (e.g., skin and lymphoma) have been reported in patients treated with calcineurin inhibitors ...”

Topical phosphodiesterase-4 (PDE4), inhibitors such as EUCRISA (crisaborole) and Opzelura (ruxolitinib), may also be used alone or in combination with corticosteroids to treat mild-to-moderate AD [58]. In the regions of AD lesions, the dermis becomes infiltrated by basophils expressing high levels of PDE4, which hydrolyzes cAMP [58, 59]. Reduced levels of cAMP promote inflammation by increasing production of IL-4. Conversely, inhibition of cAMP hydrolysis reduces inflammation by reducing production of IL-4.

Several biologics have been approved for systemic treatment of moderate-to-severe AD that is not adequately controlled by other treatments [60–64]. These are human monoclonal antibodies directed at interleukins or interleukin receptors to disrupt inflammation. They are administered by subcutaneous injection of a starter with injections every 2 weeks thereafter. Dosing frequency can be adjusted for age and weight in children.

All of the biologics have a warning of anaphylaxis. Dupixent has been associated with the occurrence of cutaneous T-cell lymphoma [65, 66]. Dupixent does not appear to be causal. Greater availability of IL-13 may promote the growth of preexisting initiated T cells.

Nemluvio is unique in that it is directed at blocking itch, and it has no infections among the adverse events [64]. All of the biologics are delivered systemically by subcutaneous injection. The properties of biologics are summarized in Table 1.

**Table 1.** Comparison of biologics for treatment of AD

Biologic	Target	Age range	Adverse effects
DUPIXENT [61] (dupilumab)	IL-4 & Il-13 receptors	6 months +	Conjunctivitis, blepharitis, herpes infections
EBGLYSS [62] (lebrikizumab)	IL-13 receptor	12 years. +	Conjunctivitis, herpes infections
Adhry [63] (tralokinumab)	IL-13	Adults	Conjunctivitis, upper respiratory tract infection
Nemluvio [64] (nemolizumab)	IL-31 receptor	12 years. +	Headache, arthralgia, urticaria, and myalgia

Several new biologics that are currently in clinical trials target the OX40 ligand on antigen presenting cells to reduce T-cell-mediated inflammation [67, 68]. In a phase 2 clinical trial, amlitelimab achieved a 75% reduction in EASI after 24 weeks. At this point some patients continued on amlitelimab while others stopped taking it. At 52 weeks, those who had stopped taking amlitelimab maintained the response seen at 24 weeks. The drug was well tolerated by those who continued taking it for 52 weeks. Rocatinlimab is in a phase 3 clinical trial which is expected to be completed in 2027 [68]. A successful phase 2 trial led to this phase 3 trial on adolescents and adults with moderate-to-severe AD.

Janus kinase (JAK) inhibitors are used for treating moderate-to-severe AD in people aged 12+ who have not responded adequately to previous therapy [69]. Examples of JAK inhibitors used in treatment of AD are RINVOQ (upadacitinib), Olumiant (baricitinib), and CIBINQO (abrocitinib). These are monoclonal antibodies in tablet form taken once per day. JAKs are tyrosine kinases that are associated with cytokine receptors. When the receptor is activated, the JAK phosphorylates a STAT protein to activate the JAK-STAT signaling pathway, leading to inflammation. Each of these JAK inhibitors has a boxed warning in its package insert warning about serious infections, lymphoma and other malignancies. The neurological signaling processes in the frontal cortex of the brain leading to itch are closely linked to inflammatory processes, and this neuronal activity can be inhibited by JAK inhibitors [70]. Further work is needed to test the clinical relevance of this finding.

A relatively new topical treatment for AD is tapinarof cream (VTAMA) [71]. Tapinarof is an aryl hydrocarbon receptor agonist which, among other things, downregulates pro-inflammatory cytokines [72]. Tapinarof also upregulated epidermal uridine phosphate ceramide glucosyltransferase, which can increase production of ceramides in the epidermis [73]. The adverse events seen in AD subjects treated topically with tapinarof cream included upper respiratory infections, folliculitis, ear infection, and influenza.

Each of these drugs alleviates AD symptoms by disrupting biochemical pathways leading to inflammation; however, the observations on infections and malignancy raise concerns about compromised immunity with some of these drugs. In addition, although the systemic biologicals produce significant improvement in AD symptoms as judged by EASI-75, approximately 50% of patients did not achieve this substantial degree of improvement after 4 months of treatment [74, 75]. It has been proposed that barrier repair therapy using a combination of topical ceramides, fatty acids, and cholesterol to repair the permeability barrier should be considered as an alternate approach [76]. Properties of the different AD treatments are summarized in Table 2.

**Table 2.** Summary of current treatments for AD

Drug	Mode of administration	Mechanism of action	Worst adverse event
Corticosteroids	Topical	Reduces production of inflammatory prostanoids	Atrophy of skin at application site
			Theoretical: allergic contact dermatitis
Calcineurin inhibitors	Topical	Reduces cytokine production by blocking T-cell replication	Skin cancer, lymphoma
			Theoretical: allergic contact dermatitis
Phosphodiesterase-4 inhibitors	Topical	Reduces inflammation by increasing cAMP	Serious infections, cancer
			Theoretical: allergic contact dermatitis
Biologics	Subcutaneous injection	Blocks inflammation/itch by binding interleukins or their receptors	Anaphylactic shock
			Theoretical: allergic contact dermatitis
Janus kinase inhibitors	Oral	Blocks the JAK-STAT inflammatory pathway	Serious infections, lymphoma, other cancers
			Theoretical: allergic contact dermatitis
EpiCeram	Topical	Occlusive barrier on skin surface	May cause temporary tingling after application
			Theoretical: allergic contact dermatitis
Tapinarof	Topical	Binds aryl hydrocarbon receptor to downregulate inflammation and upregulate glucosylceramide synthesis	Respiratory tract infections
			Theoretical: allergic contact dermatitis
pH/LOCK	Topical	Restores low pH of acid mantle and supply topical ceramides	Mild tingling at application site
			Theoretical: allergic contact dermatitis
OTC ceramide-containing moisturizers	Topical	Supplies topical ceramides	None identified
			Theoretical: allergic contact dermatitis

## EpiCeram – A Treatment Directed at the Outside-In Mechanism

EpiCeram is a medical device for treatment of AD [77]. Its marketing in the USA was authorized by the FDA in 2006. It had previously been sold as an over-the-counter moisturizer cream called TriCeram. This product is based on the theory that restoration of barrier function of the skin in AD could be obtained by topical application of a formulation containing ceramide, cholesterol, and fatty acid in high concentration (5%) with a ceramide:cholesterol:fatty acid ratio of 3:1:1 by mass [78]. This ratio is used in EpiCeram.

One study involving 121 pediatric subjects with moderate-to-severe AD showed that EpiCeram was comparable to fluticasone, a mid-strength corticosteroid [79]. Although the topical corticosteroid was more effective after 14 days, by 28 days of treatment there was no significant difference between EpiCeram and fluticasone in SCORAD, pruritus, and sleep scores.

EpiCeram was compared to a hyaluronic acid-based foam in treatment of mild-to-moderate AD [80]. By 4 weeks, both formulations had achieved statistically significant improvements, although the hyaluronic acid-based formulation was slightly better.

In another study of the efficacy of EpiCeram in AD, 24 children aged 1.5–12 years were recruited [81]. Their moisturizers were replaced with EpiCeram. Eleven of these children were already being treated with topical tacrolimus, and the other thirteen were being treated with topical steroids with or without tacrolimus. SCORAD scores improved significantly in 22 of 24 subjects by 3 weeks, and all subjects showed further improvement between 6 and 21 weeks. Improvement in TEWL paralleled reduction in SCORAD, and the ultrastructure of the intercellular spaces of the SC was found to have improved with treatment. It was concluded that a ceramide-dominant barrier repair emollient was a safe and useful adjunct in treatment of pediatric AD. This study stimulated the design of a multicenter, double-blinded clinical trial of EpiCeram, a trial to test the efficacy of EpiCeram in adult subjects, and a trial to test the efficacy of EpiCeram in prevention of AD in infants.

The plan for a study to investigate the early use of EpiCeram in infants to prevent development of AD has been published [82]. Results of a pilot study have also been published [83]. There were 40 infants enrolled in each of the EpiCeram and control groups. The infants in the EpiCeram group had EpiCeram applied to the whole body twice per day starting within the first 3 weeks. This continued for 3 months. By the end of 12 months, there was no statistically significant difference in occurrence of AD or food sensitization between the EpiCeram and control groups.

EpiCeram was compared with an over-the-counter petrolatum-based moisturizer in the treatment of pediatric subjects with mild-to-moderate AD [84]. Thirteen subjects aged 2–17 were randomly assigned to each group. Each test cream was applied three times per day. Disease severity was assessed at baseline and on days 7 and 21. There were no statistically significant differences between the two treatment groups at any time point. The over-the-counter product was at least 47 times more cost-effective than EpiCeram.

Although EpiCeram does provide some benefit, it is not more effective than an over-the-counter petrolatum-based occlusive moisturizer. The ceramide in EpiCeram is not a physiological ceramide but a pseudoceramide – hydroxypropyl bispalmitamide MEA, and the fatty acids are palmitic acid and conjugated linoleic acid. Conjugated linoleic acid is not normally found in SC. Neither fatty acid is a long-chain fatty acid as found in healthy SC. These lipids are not suitable for direct repair of the defective barrier in AD. However, the similarity to an occlusive moisturizer can be understood in that “EpiCeram forms a tri-layer on the top of the skin [85].” This would provide an occlusive layer rather than repair the barrier of the SC.

## Topical Application of Ceramides

Many over-the-counter moisturizers contain ceramides and cholesterol. These are often recommended by dermatologists. They can improve barrier function significantly as judged by reduction of TEWL; however, the reduced TEWL seen with the use of these products does not reach healthy values.

The topical use of ceramides began with a Dutch company called Gist-brocades. Gist-brocades was started to ensure an adequate supply of baker’s yeast. They eventually isolated phytosphingosine from yeast and used this to synthesize phytoceramides [86–88]. In a US patent application, Gist-brocades describes the structures of semisynthetic phytosphingosine-containing ceramides [NP], [AP], and [EOP] and potential uses of topically applied ceramide [AP] [89]. The specific ceramides described included N-stearoyl phytosphingosine (ceramide [NP](t18:0/C18:0), N-α-hydroxystearoyl phytosphingosine (ceramide [AP](t18:0/18:0) and N-(27-linoleoyloxyheptacosanoyl) phytosphingosine (ceramide [EOP](t18:0/27:0/18:2). These specific ceramide [NP] and [AP] species are the most common ceramides used in skin moisturizers. They are present in normal human SC [90–93]. However, the ceramides NP and AP from human SC contain predominantly C24:0–C26:0 fatty acids and α-hydroxy acids, respectively [90–93]. The ceramides [NP] and [AP] from human SC with C18-linked fatty acids are 12.1% and 6.1% of the total ceramide [NP] and ceramide [AP], respectively, The ω-hydroxy acids found in human ceramides [EOx] are mostly 30 carbons long or longer [90–93]. In a ceramide [EOS] fraction ω-hydroxy heptacosanoic acid represented 1.3% of the total ω-hydroxy acids.

In one experiment performed by Gist-brocades, the skin on the forearms of 15 women aged 22–43 was treated with aqueous sodium dodecyl sulfate to disrupt the barrier. Then, a formulation containing ceramide [NP] at 2 mg/mL was applied twice daily to the damaged skin. An untreated area of damaged skin and a formulation placebo-treated area of damaged skin were included as controls. After 3 days, the ceramide [NP]-treated area had recovered more than 80%. Recovery in the placebo-treated skin and untreated skin was 30% and 18%, respectively.

While the synthetic ceramides [NP] and [AP] are included among the major natural SC ceramides, the ceramides [EOx] are minor components. However, ceramides [EOx] are essential for formation of the 13 nm LPP within the intercellular lipid lamellae of the SC and serve as the precursors of the corneocyte lipid envelope [7–11, 14]. Ceramide [EOP] is included at low concentrations in some skin moisturizers.

In a lipid reconstitution study, the role of the ester-linked fatty acid in ceramide [EOS] in formation of the LPP was investigated [94]. It was found that when synthetic linoleate-containing ceramide [EOS] was substituted for natural ceramide [EOS], the LPP was still formed. With oleate-containing ceramide [EOS], the LPP formed but was not as prominent. With natural ceramide [EOS] and both of these substitutions fluid, lateral chain packing was also seen. With stearate-containing ceramide [EOS] neither the LPP nor a fluid phase was observed. It was suggested that an optimal range of fluid phase domains may be necessary for formation of the LPP [95]. In view of these findings, it was surprising that topical dispersions of stearate-containing ceramide [EOP] were equivalent to linoleate-containing ceramides [EOP] and [EOS] in restoring the LPP in detergent-damaged reconstructed human epidermis [96]. This seems likely to be attributable to lipids present in the SC of the epidermal model that promote fluid lateral chain packing. This fluidity could be provided by unsaturated fatty acids and oleate-containing cholesterol esters. These unsaturated lipids were not included in the lipid reconstitution experiments but are present in normal human SC [92, 97].

The ceramide portion of Gist-brocades eventually became part of Evonik, and Evonik has become a major supplier of ceramides for use in cosmetic products [98]. In online advertisements, Evonik has promoted several versions of a ceramide-containing product called SK-INFLUX. SK-INFLUX is a lipid concentrate for use in cosmetics. It generally contains 5 mg/mL each of cholesterol, phytosphingosine, CER[NP] and CER[AP] and 0.001 mg/mL of CER[EOP]. The recommended concentrations for usage of SK-INFLUX in cosmetic products range from 1.5% to 5% for normal, dry, and aging skin and from 3.0 to 15% for stronger protection or repair. These recommendations would result in concentrations in cosmetic products ranging from 75 µg/mL to750 µg/mL of cholesterol, phytosphingosine, CER[NP] and CER[AP] and 0.15 µg/mL–1.5 µg/mL of CER[EOP], concentrations considerably lower than those used in the Gist-brocades experiments. Some cosmetic products may contain lower concentrations of SK-INFLUX if the goal was to claim a product contained ceramides.

Another major supplier of ceramides for cosmetic products is CRODA [99]. They have a product called DS-CERAMIX-V which contains CER[NP], CER[AP], CER[EOP], phytosphingosine and cholesterol. Ceramides [NS], [AS], [NP], and [NP] are also sold individually.

Vantage Specialty Chemicals [100] sells a stereochemically correct ceramide [NdS]. This has sometimes been called ceramide [NS], even though it contains dihydrosphingosine rather than sphingosine. It has also been called ceramide [NG].

## Topical Application of Ceramides in AD

The ability of molecules to penetrate into the skin is dependent upon their lipophilicity and molecular weight [101]. The ceramides that have been used topically are highly lipophilic, which favors partitioning into the SC, but they have molecular weights greater than 500, which does not favor penetration. Successful delivery of ceramides into and through the SC has been achieved, but this is highly dependent on the formulation. The Gist-Brocades patent described oil-in-water emulsions for delivery of ceramides to skin [89]. One of these formulations was anionic while the other was nonionic, and both contained permeability enhancers. Other successful formulations include microemulsions based on phosphatidylcholines, polyglycerol fatty acid ester surfactants or TEGO CARE PL 4, which is a lauric acid ester of a glycerol polymer used as an emulsifier in cosmetics [102–104]. A microemulsion using lecithin, miglyol, and water-pentanediol exhibited excellent properties in terms of stability, capacity for ceramide [AP], and release and penetration of ceramide into the SC. Lecithin-based microemulsions proved to be superior to starch-based nanoparticles and gels for the release and penetration of ceramides isolated from oats [105]. More recent developments include the development of oil-in-water emulsions in which the ceramides are incorporated into multivesicular structures [106]. These multivesicular emulsions are thought to provide slow release of ceramide with time. One moisturizer, Atopalm, contains a pseudoceramide analog of ceramide [NP] in a multilamellar oil-in-water emulsion [107].

These creams are examples of over-the-counter ceramide-containing moisturizers that generally improve the condition of the skin in subjects with AD as judged by increased skin hydration and improvements in SCORAD, EASI, or other investigator evaluations [108–122]. Commercial formulations such as CeraVe^®^ moisturizers contain ceramide NP, ceramide AP, and ceramide EOP together with cholesterol and, sometimes, fatty acids, in a multivesicular emulsion system [108]. This approach is relevant to AD because the SC in AD shows reduced ceramide content and altered lipid organization. Studies with multivesicular emulsion ceramide formulations, including CeraVe-associated products, have shown increased SC hydration and reduced xerosis. In some studies, TEWL was reduced [109, 114, 117, 120, 122]. These findings indicate that topical ceramide-containing formulations can provide partial improvement in barrier function. However, these formulations do not fully correct the barrier abnormality in AD. Barrier function is improved, but it is not restored to values seen in healthy skin. This is consistent with the relatively low amounts of ceramides used in cosmetic formulations and with the limited penetration of high-molecular-weight lipids into the SC. In one study, TEWL of the subjects with AD was compared with TEWL from healthy control subjects [120]. The average TEWL of the eczema subjects at the beginning of this study was 16.4 g/m^2^hr. After 4 weeks of treatment, the TEWL of the subjects was reduced to 15.2 g/m^2^hr. While this represents a statistically significant reduction in TEWL compared to baseline, it is not close to the normal TEWL of 10.1 g/m^2^hr found in healthy skin. This study also showed that the chain packing of the SC lipids had become more ordered after ceramide application. This would represent a shift from hexagonal organization of untreated AD skin to orthorhombic chain packing found in healthy skin. Another study demonstrated that the topical application of a ceramide-containing moisturizer increased the ceramide content of the SC [117]. This was demonstrated by HPLC-MS analysis of lipids from tape strips of the SC. Taken together, these two studies demonstrate that topical formulations containing cosmetic levels of ceramides result in penetration of ceramides into the SC with a small improvement in lipid organization and permeability barrier function [117, 120]. It is also relevant that the ceramide classes used in these formulations do not reproduce the full ceramide profile of normal SC. In particular, they do not replace the broad range of very long-chain ceramides and acylceramides that contribute to formation of the LPP and to normal lamellar organization. Thus, these products should be regarded as adjunctive barrier-supportive treatments. They improve hydration and reduce xerosis. They do not appear to fully restore the lamellar lipid architecture required for normal SC barrier function.

The previously noted study by Gist-brocades used higher levels of ceramides to achieve restoration of barrier damage [89]. SC hydration is important for various aspects of healthy skin including physical, chemical, microbial, and immunological properties [123]. In addition to supplying ceramides and fatty acids to the SC, restoring the pH of the skin surface to a healthy pH of about 4.7 could be a significant benefit of a moisturizer [124, 125]. The low pH supports a healthy microbiome and inhibits growth of *S. aureus.* A novel cream designed to lower the skin surface pH has been shown to lower the skin surface pH of subjects with mild AD from pH 5.1 to 4.2 after 15 days of application, and this was accompanied by significant improvement in SCORAD [126]. One relatively new product for treatment of AD, pH/LOCK Eczema Relief, is based largely on restoration of the acid mantle. The pH/LOCK product has two cream formulations, 1 at pH 4.7 and 1 at pH 4.8. These creams contain colloidal oatmeal and cosmetic levels of CER[NP], CER[AP] and CER[EOP], cholesterol, and phytosphingosine. The pH of commercial moisturizers varies widely, ranging from about 3.7 through 8.2 [127].

## Conclusion

According to the outside-in mechanism for AD, restoring lipid organization in the SC should restore barrier function. This would allow resolution of inflammation. It has been demonstrated that topically applied ceramides can restore the function to a damaged barrier. However, full restoration of barrier function requires delivery of higher amounts of ceramide than are used in existing skin moisturizers. Long-chain fatty acids should also be included since these are specifically deficient in the SC of AD subjects. Cholesterol is not needed. Additional benefits would be derived from the inclusion of antimicrobial and anti-itch agents and a low pH in topical formulations.

## Acknowledgments

Conversations with and editorial suggestions from Steve Garchow were very useful in preparation of this manuscript. Nancy Wertz assisted in preparation of this manuscript.

## Conflict of Interest Statement

Philip W. Wertz was a member of the journal’s editorial board at the time of submission. Joachim W. Fluhr is an Editor of *Skin Pharmacology and Physiology*.

## Funding Sources

This study was not supported by any sponsor or funder.

## Author Contributions

Philip W. Wertz and Joachim W. Fluhr both contributed equally to the researching of literature and writing of this manuscript. Joachim W. Fluhr did the statistical analysis for Figure 4.

## Data Availability

The information presented in this review is derived from publicly available sources cited in the references.
